# Immune checkpoint molecules B7‐1 and B7‐H1 as predictive markers of pre‐eclampsia: A case–control study in a Ghana

**DOI:** 10.1002/iid3.1142

**Published:** 2024-01-10

**Authors:** Martin Awe Akilla, Ignatius Abowini Awinibuno Nchor, Moses Banyeh, Nafiu Amidu

**Affiliations:** ^1^ Department of Biomedical Laboratory Science, School of Allied Health Sciences University for Development Studies Tamale Ghana

**Keywords:** cytokines, Ghana, immune checkpoint proteins, pre‐eclampsia, pregnancy

## Abstract

**Background/Aim:**

Immune tolerance in the fetal–maternal junction is maintained by a balance in the Th1/Th2 system. Th1‐type immunity is associated with pro‐inflammatory cytokines and immune checkpoint molecules (ICMs) such as B7‐H1, while Th2‐type immunity is characterized by anti‐inflammatory cytokines and ICMs such as B7‐1. Any imbalance in the Th1/Th2 immune system may lead to adverse pregnancy outcomes such as pre‐eclampsia (PE). Hitherto, the potential of serum B7‐1 and B7‐H1 proteins as early markers of PE has not been explored in the Ghanaian population.

**Materials and Methods:**

This was a case–control study from May 2020 to April 2022 at the War Memorial and the Upper East Regional Hospitals. The study involved 291 women, including 180 (61.9%) with normotensive pregnancy and 111 (38.1%) with PE. Venous blood samples were collected and assayed for blood cell count, serum interleukins (ILs)‐4, ‐6, ‐12, ‐18, and TNF‐α as well as serum B7‐1 and B7‐H1 proteins.

**Results:**

The monocyte count (*p* = .007), the serum levels of IL‐18 (*p* = .035), TNF‐α (*p* = .001), and B7‐H1 (*p* = .006) were significantly higher in PE than in normotensive pregnancy. In addition, the monocyte count (*p* = .002), the serum levels of IL‐12 (*p* = .029), TNF‐α (*p* = .016), and B7‐1 (*p* = .009) levels were significantly higher in the third trimester than the second trimester PE. In predicting PE, the area under the curve of cytokines and ICMs ranged from 0.51 for IL‐6 to 0.62 for TNF‐α.

**Conclusion:**

PE may be characterized by a dominant Th1‐type immunity with higher levels of pro‐inflammatory cytokines and B7‐H1 proteins, but these variables may not be suitable for predicting PE.

## Graphical abstract

There are changes in serum cytokines and immune checkpoint molecules B7‐1 and B7‐H1 in pre‐eclampsia (PE). However, these changes are not predictive of PE.

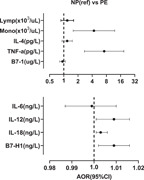

## 1 INTRODUCTION

The maternal immune system and how it is regulated are vital for the establishment and sustenance of pregnancy.^1^ Previous studies have led to the suggestion that pregnancy is indeed characterized by a state of controlled mild systemic inflammation.^2^ The maternal immune system has to recognize and tolerate the semi‐identical fetus that is carrying paternally inherited antigens as well as defend against invading pathogens.^3^ Maternal immune responses have to be balanced to prevent cytotoxic attacks on the fetus that may result in fetal loss.^4^ To achieve this, maternal immune cells, cytokines, the immune human leukocyte antigen (HLA) system, and B7 costimulatory molecules or immune checkpoint molecules (ICMs) work together to ensure normal placental and fetal development and maintenance.^5^

The maternal adaptive immune cells, including B cells, monocytes, natural killer (NK) cells, T regulatory (Tregs) cells, T helper (Th), and T cytotoxic (CTL) cells, play key roles in maintaining tolerance in the fetal–maternal junction during pregnancy.^6^ When naïve T cells encounter fetal antigens, they differentiate into T cell subsets such as T helper 1 (Th1), Th2, and Th17 cells with specific roles in maintaining immune tolerance during pregnancy.^6^, ^7^ There is usually a shift in immunity from Th1 type, during the peri‐implantation period, to a Th2‐type immunity postimplantation to attain immune tolerance.^5^ Moreover, there is a further shift from Th2‐type to Th1‐type immunity after parturition. Aside from the Th1/Th2 type immunities, the involvement of Th17‐type immunity in pregnancy has also been suggested.^5^ Uterine Tregs are known to inhibit the proliferation of Th1 and Th17 cells which also, in turn, prevent Tregs from attacking the semi‐allogeneic fetus.^7^ In addition, Tregs play a role in immune tolerance towards fetal antigens and participate in angiogenesis and spiral artery remodeling by producing IL‐10.^6^, ^7^ NK cells are another group of immune cells that participate in immune tolerance during pregnancy. Peripheral NK cells (pbNK) constitute between 10% and 15% of the total blood lymphocyte count. The uterine NK (uNK) cells play a key role in the maintenance of immune tolerance at the fetal–maternal junction through the production of cytokines and chemokines.^6^, ^7^ The pre‐pregnancy or endometrial NK (eNK) cells, a type of uNK cells, later differentiate, by the action of interleukin (ILs)‐15, into decidua NK cells (dNK). The dNK cells are pivotal in trophoblast invasion, decidualization of the endometrium, angiogenesis, and spiral artery remodeling of the placenta.^7^ Normal pregnancy is characterized by an increase and activation of monocytes.^8^ Studies have shown that there is an increase in the phagocytic activity of monocytes in pregnancy due to the presence of fetal and placental cells and other particles in maternal circulation that may lead to elevated monocyte numbers in pregnancy.^9^ There has not been a consensus as to the exact reason monocytes are activated in pregnancy. However, previous studies have suggested that monocytes are activated into pro‐inflammatory phenotypes as they interact with syncytiotrophoblast in the placental lacunae as they circulate in the blood.^10^ The HLA can stimulate an allogenic immune response by the maternal NK and T cells against the semi‐allogeneic fetus. It is therefore important to establish a maternal–fetal immune tolerance towards HLA.^11^ One of the mechanisms that establish fetal–maternal tolerance is that trophoblast express truncated HLA molecules (HLA‐G and HLA‐E) and the classical HLA Ia (HLA‐C), instead of the complete classical HLA type I (HLA‐A and HLA‐B) molecules.^6^

Th1‐type immunity involves pro‐inflammatory responses that are meant to benefit the invading trophoblast and enhance tissue remodeling as well as angiogenesis.^5^ However, later in pregnancy, Th2‐type immunity predominates which is characterized by anti‐inflammatory responses, meant to counterbalance or suppress Th1‐type immunity, at the maternal–fetal junction, to accommodate and protect placental and fetal development.^12^, ^13^ Studies have shown that Th1/Th2 immunity is associated with NK cells, HLA, specific cytokines as well as the expression of specific ICMs such as the B7 family of proteins. Any imbalance in maternal–fetal tolerance during pregnancy may lead to adverse pregnancy outcomes such as repeated fetal loss, pre‐eclampsia (PE), or preterm birth.^1^, ^12^ Th1‐type immunity is associated with cellular immunity where there is the priming of peripheral blood mononuclear cells (PBMCs) and the stimulation of circulatory syncytiotrophoblast microvesicles (STBM).^5^ Th1‐type immunity is characterized by the production of pro‐inflammatory cytokines such as IL‐12 and TNF‐α which are usually involved in immune surveillance as well as the prevention of excessive invasion of trophoblast.^14^ After implantation, naïve T cells usually differentiate into Th2‐type cells, through the participation of dendritic cells (DCs). Th2‐type cells participate in humoral immunity and may be activated by paternal antigens in the trophoblast at the fetal‐maternal junction. Th2‐type immunity is characterized by the production of anti‐inflammatory cytokines such as IL‐4 and IL‐6.^15^ The release of Th2‐type cytokines suppresses Th1 and Th17‐type immunities.^5^ The limited secretion of IFN‐γ, by low‐level IL‐18 from a monocyte, ensures Th2‐type immunity is sustained in pregnancy after implantation.^14^ This mechanism ensures allograft tolerance and normal fetal development. However, studies have shown that a predominant Th2‐type immunity may not be necessary for a successful pregnancy.^5^ There may even be a shift once again, from Th2‐type to Th1‐type immunity in late pregnancy which may arise in preparation for parturition.^14^

Aside from cytokines, ICMs such as the B7 family of proteins also participate in the immunity of pregnancy. The receptor, PD‐1 (alias, PDCD1, CD279) and its ligands, PD‐L1 (alias B7‐H1 or CD274) and PD‐L2 have been associated with Th2‐type immunity in pregnancy.^3^ The upregulation of the PD‐1/PD‐L1 axis is said to promote immune tolerance by inhibiting cytotoxic T cell activity as well as suppressing autoreactive mechanisms and enhancing T cell homeostasis in pregnancy.^14^, ^16^ Another ICM, B7‐1 (alias CTLA‐4 and CD152) and its ligands (CD80 and CD86),^1^ have been positively associated with Th1‐type immunity in pregnancy. Studies have however shown that these molecules play costimulatory functions in immune responses.^3^ The ICMs and their ligands are usually expressed on the surfaces of immune cells, such as DCs, T helper cells, NK cells, macrophages, and so on; however, the free or soluble forms of these molecules also exist in circulation and may be used as proximate measures of their expression.^3^ Previous studies have shown that B7‐H1 has both inhibitory and costimulatory effects on T cells. The inhibitory function of B7‐H1 is achieved through the signaling of program death 1 (PD‐1).^3^

The careful balance in the Th1/Th2 immunity, as well as the PD‐1/PD‐L1 and B7‐1/CD80 regulatory axis in normal pregnancy, may be disrupted in PE.^17^ Previous studies have shown that the proportions of Th1 cells and Th1/Th2 cell ratio are higher while Th2 cells are lower in PE than in the third trimester of normal pregnancy.^18^ Also, Th1‐type cells and associated cytokines such as TNF‐α, IL‐12, and IL‐18 may be elevated in PE, an indication that there is a shift to a predominant Th1‐type immunity in PE.^5^, ^19^ Similarly, the soluble forms of B7‐1 and B7‐H1 have also been associated with PE. Moreover, Th17 cells, known for their pro‐inflammatory properties, are overexpressed in PE, leading to an increase in pro‐inflammatory cytokines such as IL‐17 and IL‐22. This overexpression can potentiate the recruitment of immune effector cells and the generation of other inflammatory cytokines, contributing to chronic inflammatory conditions associated with PE,^20^ although the findings have not been universal. Cytokines and ICMs have been explored as early biomarkers for diagnosis, monitoring as well and therapy for the management of PE.^1^, ^21^ Previous studies have indicated that genetics and environment do have an effect, respectively, on the physiology and pathophysiology of pregnancy and PE. For example, the polymorphisms in the CD28 gene result in varying ligation of CD28 to its ligands, B7‐1 and B7‐2 on APCs, which may be associated with pregnancy complications.^22^ Similarly, vegetarianism has also been associated with serum levels of inflammatory biomarkers; however, the findings are inconclusive.^23^, ^24^ It is therefore necessary to conduct population‐specific studies to determine the potential changes in cytokines, B7‐H1, and B7‐1 ICMs and their predictive potential for PE in the Ghanaian population.

## 2 MATERIALS AND METHODS

### 2.1 Study design and settings

The study was case–control that was conducted from May 2020 to April 2022. This study was conducted at the Upper East Regional Hospital (UERH), Bolgatanga and the War Memorial Hospital (WMH), Navrongo. The UERH is the secondary‐level healthcare facility that serves as a referral hospital in the Upper East Region (UER) of Ghana. The hospital also renders health services to patients referred from health facilities in neighboring Burkina Faso and Togo. The WMH is a district hospital that renders health‐related services to the residents of Navrongo and neighboring communities.

### 2.2 Study population

The study involved 291 pregnant women, of whom 180 (61.9%) were normotensive pregnant while 111 (38.1%) were diagnosed with PE. Of the 180 pregnant women with normotensive pregnancy, 73 (40.6%) were in the second trimester, while 107 (59.4%) were in the third trimester of pregnancy. In addition, the women diagnosed with PE comprised 50 (45.0%) second‐trimester and 61 (55.0%) second‐trimester pregnant women. PE was defined per the guidelines of the International Society for the Study of Hypertension in Pregnancy (ISSHP) as the new onset of hypertension (systolic blood pressure ≥140 mmHg and/or diastolic blood pressure ≥90 mmHg) after 20 weeks of gestation plus proteinuria (spot urine protein at least +1 on dipstick).^25^ Women who were found to have chronic hypertension, diabetes mellitus, hepatitis, or other chronic diseases were excluded from the study.

### 2.3 Variables

The study variables included dependent and independent variables. The dependent variables were the trimester of pregnancy and the presence or absence of PE. The independent variables included sociodemographic variables (occupation, educational level, marital status, etc.), anthropometric variables (age and body mass index [BMI]), blood cell count (lymphocytes and monocytes), serum cytokines (IL‐4, IL‐6, IL‐12, IL‐18, and TNF‐α) as well as serum B‐7 proteins (B7‐1 and B‐7H1). Possible confounding variables included the age and the BMI at the time of sampling.

### 2.4 Data collection and measurements

#### 2.4.1 Socio‐demographic and anthropometric data

The socio‐demographic and obstetric data were collected using an interviewer‐administered semi‐structured questionnaire. Also, clinical data were obtained from the clinical records of the women, held at the health facility. The body weight and standing height were measured to the nearest 0.1 kg and 0.01 cm using a body scale and a stadiometer, respectively. The BMI was then calculated in kg/m^2^ following recommended guidelines.^26^

#### 2.4.2 Laboratory analysis

A single venous blood sample was collected and dispensed into K_3_EDTA anticoagulant and gel‐separator vacutainer tubes. A full blood count (FBC) analysis was performed using the anticoagulated blood sample on a five‐part automated hematology analyzer (Sysmex America, Inc) within an hour after the blood draw. The blood in the gel‐separator tube was allowed to clot before centrifugation to obtain serum. The serum samples were aliquoted in duplicates and then stored at −20°C before analysis. The serum levels of IL‐4, IL‐6, IL‐18, TNF‐α, B7‐1, and B7‐H1 were determined using the enzyme‐linked immunosorbent assay technique.

#### 2.4.3 Statistical analysis

The data were collected onto an Excel Spreadsheet and then analyzed using SPSS (v26), GraphPad Prism (v8), and MedCalc (v14.8.1.0) statistical packages. The Shapiro‐Wilk test was used to test for the normality of the data and check for outliers. Extreme outliers were removed and then replaced with the group's series mean value by imputation. Categorical variables were summarized as frequencies (percent), while continuous variables were summarized as median (interquartile range). The differences in the distribution of data between two groups were determined using nonparametric Mann–Whitney *U* test. Multivariable binary logistic regression analyses were performed to determine the association between a dependent or reference variable and predictor or other variables while adjusting for covariates. The odds ratios with their corresponding 95% confidence intervals from regression analyses were then presented as forest plots. The predictive abilities of the independent variables for PE were determined using the receiver operator characteristic test curve, selecting the Hanley and McNeil technique.^27^ All statistical analyses were two‐tailed at a significance level of *p* < .050. All findings were reported following The Strengthening the Reporting of Observational Studies in Epidemiology guidelines.

## 3 RESULTS

### 3.1 Sociodemographic and obstetric characteristics of the study population

The distribution of the study population did not significantly differ by marital status, educational status, occupation, the pregnancy trimester, or the offspring sex at birth (Table 1). However, the distribution by their cultural affiliation was significantly different (*χ* ^2^ = 11.729, *p* = .003).

**Table 1:** Sociodemographic and obstetric characteristics of the study population.

Variable	NP (*n* = 180)	PE (*n* = 111)	*χ* ^2^, *df*	*p* Value
Cultural affiliation				
Mole‐Dagomba	171 (95.0)	92 (82.9)	11.729, 2	.003
Akan	4 (2.0)	7 (6.3)		
Others	5 (2.8)	12 (10.8)		
Marital status			1.710, 2	.425
Married	174 (96.7)	107 (96.4)		
Co‐habitation	4 (2.2)	4 (3.6)		
Single	2 (1.1)	0 (0.0)		
Occupation			1.019, 2	.601
Unemployed	28 (15.6)	19 (17.1)		
Self‐employed	92 (51.1)	50 (45.0)		
Salary	60 (33.3)	42 (37.8)		
Educational status			4.541, 3	.209
None	5 (2.8)	4 (3.6)		
Basic	57 (31.7)	38 (34.2)		
Secondary	52 (28.9)	20 (18.0)		
Tertiary	66 (36.7)	49 (44.1)		
Offspring sex at birth			1.828, 1	.183
Female	73 (40.6)	54 (48.6)		
Male	107 (59.4)	57 (51.4)		
Pregnancy trimester			0.567, 1	.466
Second	73 (40.6)	50 (45.0)		
Third	107 (59.4)	61 (55.0)		

*Note*: The results are presented as frequency (percent).

Abbreviations: NP, normotensive pregnant; PE, pre‐eclampsia.

### 3.2 Comparison of variables between normotensive pregnancy and PE

**Figure 1:**
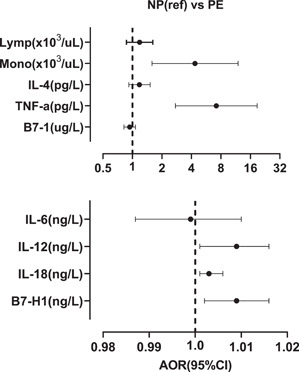
Forest plot showing the adjusted odds ratios (AORs) and 95% confidence intervals (95% CIs) of blood cells, cytokines, and B7 protein levels in pre‐eclampsia (PE) regarding normotensive pregnancy (NP). The binary logistic regression analyses were adjusted for age and cultural affiliation.

The anthropometric, hematological, and biochemical variables of the study population for normotensive pregnancy and PE were compared (Table 2). The blood monocyte count (*p* = .007), the serum IL‐18 (*p* = .035), TNF‐α (*p* = .001), and B7‐H1 (*p* = .006) levels were significantly higher in PE than in normotensive pregnancy. However, the participants in the PE group were significantly older than those in the normotensive group (*p* = .005). The comparison was therefore adjusted for age and cultural affiliation in a binary logistic regression analysis (Figure 1). The participants in the PE group were more likely to have higher monocyte count, serum IL‐18, TNF‐α, and B‐7H1 than those with normotensive pregnancy.

**Table 2:** Comparison of the anthropometric, hematological, and biochemical variables between normotensive pregnancy (NP) and pre‐eclampsia (PE).

Variable	NP	PE	*p* Value
Age (years)	29.0 (26.0–33.0)	31.0 (27.0–35.0)	.005
BMI (kg/m^2^)	27.9 (24.2–31.5)	27.7 (23.8–29.7)	.442
LYMP ×10^3^/µL	1.5 (1.2–1.8)	1.6 (1.0–1.9)	.401
MONO ×10^3^/µL	0.5 (0.4–0.7)	0.6 (0.4–0.8)	.007
IL‐4 (pg/L)	2.5 (1.2–3.2)	2.4 (2.1–3.0)	.373
IL‐6 (ng/L)	71.3 (57.4–85.0)	72.8 (52.9–85.2)	.897
IL‐12 (ng/L)	148.6 (123.2–349.3)	152.2 (121.0–190.2)	.066
IL‐18 (ng/L)	290.0 (202.2–349.3)	309.2 (248.4–362.2)	.035
TNF‐α (pg/L)	0.9 (0.8–1.1)	1.0 (0.9–1.3)	.001
B7‐1 (µg/L)	5.5 (4.0–7.1)	4.9 (4.0–6.7)	.448
B7‐H1 (ng/L)	87.5 (59.3–118.7)	110.0 (79.1–130.2)	.006

*Note*: The results are presented as median (interquartile range). The distribution of the data between groups was compared using the Mann–Whitney *U* test (unpaired, two‐tailed).

### 3.3 Comparison of second and third‐trimester variables in normotensive pregnancy

The second and third‐trimester variables in normotensive pregnancy were compared (Table 3). The participants did not significantly differ in age and BMI. However, the monocyte count was significantly higher in the second than the third (*p* = .002), while the serum IL‐6 was significantly higher in the third than the second (*p* < .001) trimester of pregnancy.

**Table 3:** Comparison of the anthropometric, hematological, and biochemical variables by trimesters (Ts) in normotensive pregnancy (NP).

Variable	2TNP	3TNP	*p* Value
Age (years)	29.0 (26.5–32.0)	28.0 (26.0–34.0)	.757
BMI (kg/m^2^)	27.3 (23.8–30.9)	28.1 (24.6–32.0)	.347
LYMP ×10^3^/µL	1.5 (1.2–1.7)	1.5 (1.2–1.9)	.466
MONO ×10^3^/µL	0.6 (0.4–0.8)	0.5 (0.3–0.6)	.002
IL‐4 (pg/L)	2.1 (1.1–3.1)	2.5 (1.6–3.2)	.503
IL‐6 (ng/L)	63.1 (51.8–74.9)	77.8 (61.9–85.2)	<.001
IL‐12 (ng/L)	148.3 (121.0–172.4)	148.8 (125.2–165.1)	.813
IL‐18 (ng/L)	250.3 (192.8–344.4)	311.9 (210.9–350.8)	.064
TNF‐α (pg/L)	0.9 (0.9–1.2)	0.9 (0.8–1.0)	.074
B7‐1 (µg/L)	6.0 (4.0–7.4)	5.3 (3.9–6.9)	.153
B7‐H1 (ng/L)	85.7 (59.0–107.3)	104.1 (58.8–122.3)	.102

*Note*: The results are presented as median (interquartile range). The distribution of the data between groups was compared using the Mann–Whitney *U* test (unpaired, two‐tailed).

### 3.4 Comparison of second and third‐trimester variables in PE

**Figure 2:**
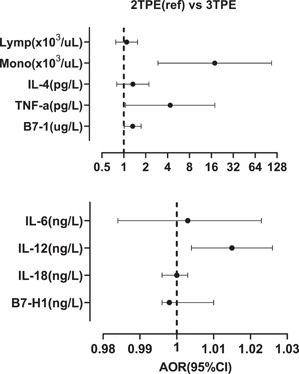
Forest plot showing the adjusted odds ratios (AORs) and 95% confidence intervals (95% CIs) of blood cells, cytokines, and B7 protein levels in third‐trimester pre‐eclampsia (3TPE) regarding second‐trimester pre‐eclampsia (2TPE). The binary logistic regression analyses were adjusted for body mass index.

The anthropometric, hematological, and biochemical variables of the study population between the second and the trimester PE were compared (Table 4). The blood monocyte count (*p* = .002), the serum IL‐12 (*p* = .029), TNF‐α (*p* = .016), and B7‐1 (*p* = .009) levels were significantly higher in PE that occurred in the third than the second trimester of pregnancy. However, the BMI of the participants in the second‐trimester group was significantly higher than those in the third‐trimester group (*p* = .001). The comparison was therefore adjusted for BMI in a binary logistic regression analysis (Figure 2). The third‐trimester PE group was more likely to have higher monocyte count, serum IL‐12, TNF‐α, and B‐71 than the second‐trimester PE group.

**Table 4:** Comparison of anthropometric, hematological, and biochemical variables in pre‐eclampsia (PE) by pregnancy trimester (T).

Variable	2TPE	3TPE	*p* Value
Age (years)	32.0 (28.0–36.0)	31.0 (26.0–35.0)	.335
BMI (kg/m^2^)	29.3 (24.4–32.8)	25.0 (23.1–28.6)	.001
LYMP ×10^3^/µL	1.6 (1.2–24.4)	1.6 (1.0–2.0)	.808
MONO ×10^3^/µL	0.5 (0.4–0.7)	0.7 (0.5–0.9)	.002
IL‐4 (pg/L)	2.4 (1.9–2.6)	2.5 (2.2–3.0)	.164
IL‐6 (ng/L)	69.0 (52.9–82.4)	75.2 (57.9–85.6)	.502
IL‐12 (ng/L)	152.2 (121.0–164.6)	167.4 (123.0–210.3)	.029
IL‐18 (ng/L)	303.6 (250.3–344.4)	312.1 (242.5–375.0)	.788
TNF‐α (pg/L)	0.9 (0.9–1.3)	1.2 (0.9–1.4)	.016
B7‐1 (µg/L)	4.9 (4.0–5.8)	6.1 (4.4–7.1)	.009
B7‐H1 (ng/L)	105.1 (79.1–135.5)	110.0 (73.2–129.6)	.801

*Note*: The results are presented as median (interquartile range). The distribution of the data between the groups was compared using the Mann–Whitney *U* test.

### 3.5 Predicting PE with cytokines and B7 molecules

**Figure 3:**
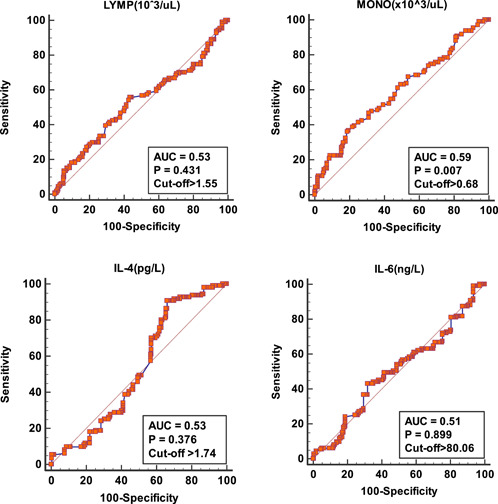
Receiver operator characteristics curves showing the comparative ability of blood lymphocyte count (LYMP), monocyte count (MONO), interleukin (IL)‐4, and IL‐6 in predicting pre‐eclampsia. AUC, area under the curve.

**Figure 4:**
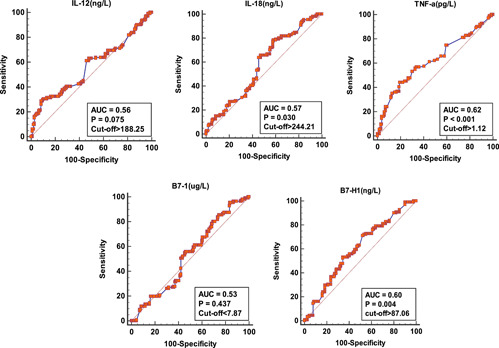
Receiver operator characteristics curves showing the comparative ability of serum interleukin (IL)‐6, IL‐12, IL‐18, TNF‐α, B7‐1, and B7‐H1 in predicting pre‐eclampsia. AUC, area under the curve.

The predictive abilities of the variables for PE were assessed using the hematological and biochemical variables of women with normotensive pregnancy and PE in the second and third trimesters of pregnancy (Table 5 and Figures 3 and 4). The total monocyte count, serum IL‐18, TNF‐α, and B7‐HI levels were predictive of PE albeit poorly. The area under the curve (AUC) ranged from 0.51 to 0.62. TNF‐α was most predictive of PE with an AUC of 0.62, while IL‐6 was the least predictive of PE (AUC = 0.51). The recommended criterion of TNF‐α for predicting PE was determined at >1.12 pg/L with a sensitivity and specificity of 44.1% and 80.6%, respectively.

**Table 5:** Comparative ability of blood cells, cytokines, and B7 proteins in predicting pre‐eclampsia (PE).

Variable	Sensitivity (95% CI)	Specificity (95%CI)	+LR (95%CI)	−LR (95%CI)	Cutoff	AUC
LYMP ×10^3^/µL	55.9 (46.1–65.3)	56.7 (49.1–64.0)	1.3 (1.0–1.6)	0.8 (0.6–1.0)	>1.55	0.53
MONO ×10^3^/µL	36.9 (28.0–46.6)	80.6 (74.0–86.1)	1.9 (1.3–2.8)	0.8 (0.7–0.9)	>0.68	0.59
IL‐4 (pg/L)	91.0 (84.1–95.6)	33.9 (27.0–41.3)	1.4 (1.2–1.6)	0.3 (0.1–0.5)	>1.74	0.53
IL‐6 (ng/L)	43.2 (0.1–0.5)	68.3 (61.0–75.1)	1.4 (1.0–1.8)	0.8 (0.7–1.0)	>80.06	0.51
IL‐12 (ng/L)	28.8 (20.6–38.2)	91.7 (86.6–95.3)	3.5 (2.0–6.1)	0.8 (0.7–0.9)	>188.25	0.56
IL‐18 (ng/L)	78.4 (69.6–85.6)	41.7 (34.4–49.2)	1.3 (1.1–1.6)	0.5 (0.3–0.8)	>244.21	0.57
TNF‐a (pg/L)	44.1 (34.7–53.9)	80.6 (74.0–86.1)	2.3 (1.6–3.3)	0.7 (0.6–0.8)	>1.12	0.62
B7‐1 (µg/L)	95.5 (89.8–98.5)	16.1 (11.1–22.3)	1.1 (1.1–1.2)	0.3 (0.1–0.7)	≤7.87	0.53
B7‐H1 (ng/L)	71.2 (61.8–79.4)	47.8 (40.3–55.3)	1.4 (1.1–1.6)	0.6 (0.4–0.8)	>87.06	0.60

Abbreviations: AUC, area under the curve; CI, confidence interval; LR, likelihood ratio.

## 4 DISCUSSION

The study sought to predict PE using serum levels of B7‐1 and B7‐H1 proteins. After controlling for covariates, the blood monocyte count, the serum IL‐12, IL‐18, TNF‐α, and B7‐H1 protein levels were significantly higher in PE than in normotensive pregnancy. Moreover, the blood monocyte count, the serum IL‐12, TNF‐α, and B7‐1 protein levels were significantly higher in PE occurring in the third than in the second trimester of pregnancy. The total monocyte count, serum IL‐18, TNF‐α, and B7‐HI levels were predictive of PE albeit poorly.

The total monocyte count was significantly higher in PE than in normotensive pregnancy. In addition, PE occurring in the third trimester of pregnancy was significantly associated with a higher monocyte count than in the second trimester. These findings are consistent with previous studies.^8^, ^28^, ^29^ PE is characterized by systemic inflammation with the associated pro‐inflammatory cytokines. Monocytes are stimulated and attracted to inflammatory sites by pro‐inflammatory cytokines to engage in phagocytic activities.^29^ In addition, members of the immunoglobulin superfamily, in inflammatory states such as PE, stimulate endothelial cells and macrophages to secrete monocyte chemoattractant protein 1 (MCP‐1), which enhances the further recruitment of monocytes.^29^

The serum pro‐inflammatory cytokines IL‐12, IL‐18, and TNF‐α were significantly increased in PE. Also, women with PE were more likely to have higher levels of IL‐12 and TNF‐α in the third trimester and IL‐18 in the second trimester of pregnancy when compared to controls. These findings are consistent with those of previous studies.^30^, ^31^ While reports on the utility of IL‐12 and TNF‐α as early biomarkers of PE are mixed, previous studies showed that second‐trimester IL‐18 levels were higher in women who later develop PE when compared to controls.^21^, ^32^ While IL‐18 alone can stimulate Th1‐type immune responses, in the presence of IL‐12, IL‐18 tend to induce Th2‐type immune responses.^33^ Consistent with the findings of this study, a review of previous studies has shown that TNF‐α tends to be elevated in the third trimester in PE than controls, albeit similar studies have reported no significant difference or even lower levels of TNF‐α in PE.^21^ It has, however, been shown that in two of these previous studies that reported no significant difference in TNF‐α levels in PE or lower levels than controls were characterized by either smaller sample sizes (*n* < 40)^34^, ^35^ or the authors did not control for confounding variables such as smoking, urinary or respiratory infections.^34^, ^36^ In the third study, the sample size was relatively smaller (cases = 24; controls = 31), and the sampling period overlapped between the second and third trimesters of pregnancy.^35^ Contrary to the current study, previous studies have found IL‐18 to be elevated in the third, not the second, trimester of pregnancy.^21^, ^37^ It has been explained that, unlike normal pregnancy where there is a shift from Th1‐type to Th2‐type immunity as pregnancy progresses, in PE, this shift is lacking resulting in a predominant Th1‐type immunity.^19^ Th1‐type immunity is characterized by increased secretion of pro‐inflammatory cytokines such as IL‐12, TNF‐α, and IL‐18.^15^, ^38^

In this study, B7‐H1 was significantly higher in PE, which. was consistent with a previous study.^39^ The PD‐1/PD‐L1 system may be altered in PE to augment the immune response even though the mechanism is not well understood.^40^ In one previous study, while there was an increased expression of B7‐H1 on NKT‐like cells in early‐onset PE, suggestive of the involvement of the PD‐1/PD‐L1 axis in early‐onset PE, no significant change in the soluble or free form of B7‐H1 was found. This observation made the authors doubt the involvement of soluble B7‐H1 in the pathophysiology of early‐onset PE.^41^ Studies have shown that there is a significant reduction in the function of T regulatory cells (Tregs) and an increased expression of PD‐1 on exhausted Tregs in PE. The presence of PD‐1 on exhausted Tregs may indicate that PD‐1 expression on Tregs may lead to function reduction which may contribute to PE.^40^

When assessed for predictive ability, TNF‐α was the most predictive, while IL‐6 was the least predictive of PE. However, none had a better diagnostic potential as all the AUCs were less than 0.65. The diagnostic ability of cytokines and B7 proteins for PE have been mixed. This observation may stem from meta‐analytic studies that were inconsistent in their findings as to whether cytokines and B7 proteins are elevated or reduced in PE.^42^, ^43^ The variabilities in findings may be due to differences in genetic, environmental factors, or methodology.

The current study has some strengths. While the study of cytokines levels in pregnancy and PE is not uncommon in Africa,^44^, ^45^, ^46^ the determination of changes in B7 proteins in pregnancy and PE is rare. Also, the effect of confounding variables such as age and BMI were controlled. The authors, however, acknowledge that a longitudinal study involving multiple sampling of the same study population would have shown the trend of cytokines and B7 proteins as the pregnancy or PE progresses. Future studies should take into account the limitations of the current study.

## 5 CONCLUSION

The study observed that the levels of IL‐12, IL‐18, TNF‐α, and B7‐H1 were significantly higher in PE than in normotensive pregnancy. PE may be characterized by a dominant Th1‐type immunity with systemic inflammation and increased production of pro‐inflammatory cytokines and B7‐H1 immune checkpoint proteins. The monocyte count, IL‐18, TNF‐α, and B7‐HI were predictive of PE albeit poorly. These results should be validated using another cohort in the same population.

## AUTHOR CONTRIBUTIONS

**Martin Awe Akilla**: Conceptualization; data curation; formal analysis; investigation; methodology; writing—original draft; writing—review and editing. **Ignatius Abowini Awinibuno Nchor**: Investigation; Writing—review and editing. **Moses Banyeh**: Formal analysis; supervision; writing—original draft; writing—review and editing. **Nafiu Amidu**: Conceptualization; methodology; project administration; supervision; writing—review and editing.

## CONFLICT OF INTEREST STATEMENT

The authors declare no conflict of interest.

## ETHICS STATEMENT

The study was conducted following the recommended guidelines for human subject studies as stipulated by the Declaration of Helsinki (1964) or its later amendments. The study was approved by the institutional review board of Navrongo Health Research Center (NHRC) with the ethical approval number: NHRCIRB378. Written informed consent was obtained from all the women before they were included in the study. Participation in the study was voluntary and the participants could opt‐out at any stage of the study.

## Data Availability

The data supporting these findings can be obtained from the corresponding author upon a reasonable request.
